# Outlines of Comparative Anatomy, Presenting a Sketch of the Present State of Knowledge, and of the Progress of Discovery in That Science; and Designed to Serve as an Introduction to Animal Physiology, and to the Principles of Classification in Zoology

**Published:** 1842-01

**Authors:** 


Art. III.—*Outlines of Comparative Anatomy, presenting a Sketch of the present state of knowledge, and of the progress of discovery in that science; and designed to serve as an Introduction to Animal Physiology, and to the Principles of Classification in Zoology.* By Robert E. Grant, m.d. f.r.s. l. & e. f.l.s. &c. &c., Professor of Comparative Anatomy in University College, London.—*London*, 1841. 8vo, pp. 656.

We scarcely know whether we are to regard this work as now complete, according to the views of the author and publisher, or whether more of it is still to appear, at some period equally indefinite with that of the publication of the several parts of which it consists. We should gladly believe that the volume before us does not complete the work, since it does not include one of the most interesting and important departments of the science—the generative system; and, moreover, it is destitute of introduction, table of contents, index, or any other guide to facilitate its use. From the manner in which it is put forth, however, we are bound to infer that it must be regarded, for the present at least, as complete; and our remarks upon it will, therefore, be made with this understanding. We hope on another occasion to lay before our readers a full account of this important volume; at present we purpose giving a mere note of its general character and contents.

We extremely regret to be obliged to speak of the work in what may seem a depreciating spirit; but we feel it to be our duty to enter our protest against the mode in which it has been published; and the more so because there are at the present time several important works in the course of publication to which the same remarks equally apply. It has come forth in seven parts, of which the *first* was published early in 1835, with the following announcement: "These Outlines will appear in Four Parts, in order to accelerate the publication. The succeeding parts will appear at regular intervals, so as to complete the work by the 1st of October next." The *second* part was published in July 1835, with the announcement that the work would be completed "in the course of next winter." The *third* part did not come out until June 1836; and it was then stated that the work would be completed in Five Parts, and that the next would be published in the following October. The promise was again broken; the *fourth* part did not appear until 1837. The *fifth* part was delayed until 1839; and was far from completing the work. A *sixth* was published in 1840; and a *seventh* has just made its appearance. Now we hold that any author who thus breaks his faith with the public (even from unavoidable causes, as we hope may have been the case in the present instance), inflicts a grievous injury both upon them and upon his brethren of the craft; upon them, by leading them to purchase, under the idea of their being speedily completed, portions of works which, whilst incomplete, are of comparatively little use to them, and thus preventing them from expending their money upon others which, with perhaps less intrinsic merit, will be of more immediate value to them; and upon his brethren, by inducing a general distrust in any such promises, even when a fair guarantee can be given that they shall be fulfilled. Further, we are confident that the mischief falls back, in the end, upon the author himself; for the confidence of the public will be most assuredly withheld from any work in which he may hereafter engage, until it shall be completed; and then much of its novelty will be lost. We can scarcely imagine a case in which there could be less excuse for the delay than there seems to us to have been in the present; for the volume before us is little else than an abridgment of the course of lectures reported in the Lancet (to the accuracy of which reports Professor Grant himself bore testimony), of which the publication was completed about the same time with the appearance of the first part. Upon an attentive examination of the work, we can find very little that is not contained in those lectures. Indeed, if it had been Professor Grant's desire that the whole should represent the state of science in the year 1835, instead of its advanced condition in the year 1841, he could scarcely have excluded more completely the results of late enquiries. From this remark, however, we must exempt the last part, which contains an account of the Tegumentary Organs, based upon the recent discoveries of Henle, Schwann, and others. We feel bound to notice a serious error into which Professor Grant has fallen, in his description of these parts. He has continually used the term *cytoblast* as synonymous with "nucleated cell," instead of employing it, according to the meaning of its inventor, to designate the *nucleus* of the cell. We should not have adverted to this, but for the apprehension that it may prove a source of great perplexity to the readers of the treatise.

We have made the foregoing remarks under a painful sense of our duty to the public; and we now come to discharge the much more gratifying task of strongly recommending the work to the notice of our readers. In the opinion we express of it as a whole, we shall, for the reason assigned above, consider ourselves as reviewing it at the period at which it was originally intended to have been completed, namely October, 1835. At that time it would have certainly stood without a rival among British treatises on Comparative Anatomy; in regard alike to the comprehensiveness of its plan, the philosophical spirit which pervades it, the fulness of the details into which the reader is led without weariness, and the graphic truth of the descriptions. Professor Grant has long been known to the scientific world by his unwearied devotion to his favorite pursuit; and we know many who feel pleasure in attributing to him much of their taste for it. Of two out of three English works, which are at present in the hands of the student of comparative anatomy and physiology, we know that the authors are largely indebted to him for their materials; and we only regret that the tardiness of the completion of his own treatise should have prevented it (as we fear it must) from taking that place in public estimation to which it is justly entitled. We believe that Dr. Grant was the first, in this country, who attempted (in his lectures) to give a systematic exposition of the several organs of the animal body, in the varied, yet progressively advancing forms, which they present when traced upwards through the ascending scale. This exposition was characterized by a strongly-marked originality, in combination with a thorough acquaintance with the labours of others; it was full and clear in its details, yet not too minute to be impressed forcibly on the mind; and these details were connected by principles of which he never allowed his audience to lose sight, although they were not enunciated with the pompous dogmatism of some foreign *savans*. These qualities are strongly marked in the treatise before us; and notwithstanding the disadvantages under which it *now* appears, we can still recommend it as containing what we cannot point to in any other English work—a full and graphic description of the several systems of the animal body, each considered systematically by itself, yet in connexion with the rest. It is illustrated by 147 wood-engravings, many of them containing several subjects, and on the whole well executed, though rather inferior in style to the highly-finished illustrations of more recent works. We may as well subjoin a list of the heads of the treatise, which are as follows: I. Organs of Support, or Osseous System; II. Organs of Attachment, or Ligaments; III. Muscular System, or Active Organs of Motion; IV. Nervous System, or Organs of Sensibility and Motility; V. Organs of the Senses; VI. Organs of Digestion; VII. Chyliferous System; VIII. Sanguiferous System; IX. Organs of Respiration; X. Organs of Secretion; XI. Lymphatic System; XII. Excreting Organs; XIII. Tegumentary Organs.

